# Tackling Diabetes Through Translation

**DOI:** 10.4103/0970-0218.66853

**Published:** 2010-04

**Authors:** Badrinarayan Mishra

**Affiliations:** Rural Medical College, PIMS, PMT Loni Bk. 413736, Rahata, Ahmednagar, India E-mail: badrinmishra@gmail.com

To improve human health, scientific discoveries must be translated into practical applications. The process of translation from bench to bedside and from there to the community at large is known as translational research. Thus translational research occurs through two phases.

In phase one, translation discoveries typically begin at ‘the bench,’ with basic research, in which scientists study disease at a molecular or cellular level. Then it progresses to the clinical level, or the patient’s ‘bedside’. Scientists are increasingly aware that this ‘bench-to-bedside’ approach to translational research is really a two-way street. Basic scientists provide clinicians with new tools for use in patients and for assessment of their impact, and clinical researchers make novel observations about the nature and progression of disease that often stimulate basic investigations.

Phase two translation promotes the adoption of promising clinical research by community-based healthcare systems under uncontrolled and often uncontrollable conditions. This includes effectiveness research, dissemination and implementation research, and policy research.(1)

Effectiveness research determines whether findings from the efficacy studies are applicable in typical community settings.

Dissemination and implementation research tests are strategies to implement effective interventions more widely. Policy research examines whether an intervention is worth implementing in practice. (Other terms also exist including quality improvement research, knowledge translation, and diffusion of innovations).

The translational program encourages cross-fertilization of ideas between disciplines. In case of diabetes there is an increasing recognition of the type 1 and type 2 diseases. The cardiovascular and other complications of these may converge on common pathophysiological processes, such as, the development of insulin resistance, inflammation, oxidative stress, and alterations in the central and peripheral neural activity. Hence the need for translational research activities is well pronounced. Several large, controlled, clinical trials have established ‘gold standard’ approaches for treating type 1 and type 2 diabetes, and for preventing type 2 diabetes in individuals at high-risk for developing the disorder.

The difficulties inherent in achieving good glucose control and preventing diabetes complications make prevention a compelling strategy; this is particularly true for type 2 diabetes, which is clearly linked to modifiable risk factors, for example, obesity and a sedentary lifestyle. The Diabetes Prevention Program (DPP) was designed to prevent or delay the development of type 2 diabetes in individuals at high risk for its development, by virtue of their having impaired glucose tolerance (IGT). The study results have been reported recently (2) and demonstrated that an intensified lifestyle or drug intervention in individuals with IGT, prevented or delayed the onset of type 2 diabetes. The results were striking. Lifestyle intervention reduced diabetes incidence by 58% and the drug metformin reduced it by 31% compared to the placebo. The effects were similar for men and women and for all racial and ethnic groups. Similar effects of lifestyle intervention were seen in another recent study conducted in Finland. Cost-effective strategies for promoting lifestyle modification in high-risk individuals, outside the setting of a controlled, clinical trial, need to be established. Population-based, as well as generalizable, clinic-based strategies are needed to establish cost-effective programs, to identify individuals at high-risk who could benefit from prevention programs, and/or successfully promote lifestyle change.

Childhood obesity, the prevalence of which has more than doubled in the past two decades, is a major risk factor for type 2 diabetes. Indeed, the increase in childhood obesity has been linked to an alarming rise in type 2 diabetes in the pediatric population. Family-based behavioral interventions have been shown to have a long-term impact on the degree of overweight.( 3) However, cost-effective interventions in primary care and community-based settings are needed.

In addition, while behavioral treatment of obesity in adults leads to clinically significant weight loss, prevention of weight regain remains an elusive goal for many. Continuing care models show promise in promoting long-term weight maintenance (4) and cost-effective means of providing such care need to be developed.

Finally, the results of the ongoing clinical trials that also address the prevention and/or treatment of type 1 or type 2 diabetes and obesity are likely to become available in the near future. (5) In the event of positive outcomes in any of these studies, it is even more crucial that effective translation strategies be developed and adopted, to improve adherence to the accepted standards of diabetes care, and to overcome the barriers of translation of scientific advances into clinical practice.

There are common barriers, which impede the adoption and implementation of current knowledge in the process of translation of a new science to patient care. The prominent ones are healthcare provider’s knowledge, communication between patient and healthcare provider, attitudes and beliefs of the patient, and the community and its culture. Other important barriers are racial and ethnic disparities, variations in settings including the healthcare system, clinical traditions, socio-economic status, and cost. Therefore, the proposed research studies should be designed to overcome these barriers.

From India’s point of view a lot more has to be done. The diabetes capital of the world should lead the field of translational research, pertaining to the disease. (5) This can happen by developing and adopting proven preventive strategies that promote the adoption of healthy lifestyles, which will reduce obesity and diabetes. Similarly research strategies that enhance glycemic, blood pressure, and lipid control, or reduce risk factors for the development of the complications of type 1 or type 2 diabetes should be promoted. We should draft and work on interventions that enhance long-term maintenance of weight loss and prevention of weight regain after initial weight loss. which would work within the limitations of existing healthcare delivery system and patient demography. Studies designed to determine the role of patient/provider communication on diabetes outcomes, and strategies to facilitate such communication, should find favor. Strategies to enhance patient or provider education and studies on information technology and decision-support, to facilitate evidence-based prevention and management have also a crucial role to play. Finally, the testing of community-based programs to provide education and behavior modification at lower cost, and studies that test interventions to treat childhood and adolescent obesity in primary care or community settings must become the front runners.

Successful implementation of the above-mentioned topics can do a world of good to Indian diabetics.
